# Correlation Between Serum Amisulpride Concentration, Therapeutic Efficacy, and Glycolipid Metabolism in the Treatment of Adult Female Schizophrenia

**DOI:** 10.62641/aep.v53i4.1806

**Published:** 2025-08-05

**Authors:** Yajun Lin, Weidong Xu, Lulu Yu, Yingying Chen

**Affiliations:** ^1^Department of Laboratory, The Third People’s Hospital of Yongkang, 321300 Yongkang, Zhejiang, China; ^2^Ward 4, The Third People’s Hospital of Yongkang, 321300 Yongkang, Zhejiang, China; ^3^Department of Laboratory, Yongkang Hospital, 321399 Yongkang, Zhejiang, China

**Keywords:** schizophrenia, amisulpride, blood drug concentration, lipid metabolism, correlation

## Abstract

**Background:**

Amisulpride is a novel atypical antipsychotic (AAP) with slower absorption, metabolism, and excretion in females, potentially leading to elevated plasma concentrations. This study aimed to explore the correlation between serum amisulpride levels and therapeutic efficacy, glycolipid metabolism and side effects in adult female patients with schizophrenia (SCH).

**Methods:**

A retrospective study was conducted involving 122 adult female SCH patients admitted to the Third People's Hospital of Yongkang between January 2020 and January 2022. Fasting venous blood samples were collected at baseline and 1, 2, 4 and 8 weeks post-treatment with amisulpride. Key parameters measured included serum amisulpride concentration, Brief Psychiatric Rating Scale (BPRS) scores, fasting blood glucose, total cholesterol (TC), triglycerides (TG), high-density lipoprotein (HDL), low-density lipoprotein (LDL), and side effect scores.

**Results:**

Serum amisulpride levels significantly increased at 2, 4, and 8 weeks compared to the first week (*p* < 0.05), while BPRS scores significantly decreased at all time points compared to those before treatment (*p* < 0.05). A strong negative correlation was observed between amisulpride concentration and BPRS scores (r = –0.948, *p* < 0.001). Significant alterations in fasting blood glucose, TC, TG, HDL, and LDL levels were observed post-treatment (*p* < 0.05). Serum amisulpride concentration negatively correlated with fasting blood glucose, TC, and LDL (r = –0.622, –0.160, –0.796, respectively, *p* < 0.001) and positively correlated with TG (r = 0.447, *p* < 0.001). Side effects scores increased significantly after 2, 4, and 8 weeks compared to the first week (*p* < 0.05), with amisulpride concentration positively correlating with side effects scores (r = 0.739, *p* < 0.001).

**Conclusion:**

Serum amisulpride levels in female SCH patients are closely correlated with therapeutic efficacy, glycolipid metabolism and incidence of side effects, respectively. Monitoring serum concentrations may provide valuable insights for guiding personalized medication management and optimize treatment outcomes.

## Introduction

Schizophrenia (SCH) is a chronic mental disorder that often manifests in young adults, with women typically experiencing symptoms onset later in life and presenting milder symptoms than men [1, 2]. Due to slower drug absorption, metabolism, and excretion in females, plasma concentrations of antipsychotic medications tend to be higher, increasing the risk of side effects [3]. Therefore, in recent years, there has been growing attention to the efficacy and side effect profiles of antipsychotic drugs in female patients.

Amisulpride, a novel atypical antipsychotic (AAP), is a highly selective dopamine D2 and D3 receptor antagonist [4]. Its primary pharmacological mechanism is the selective inhibition of dopamine receptors [5]. Amisulpride has proven effective in alleviating symptoms in patients with first-episode schizophrenia [6]. As an AAP, it is frequently used in the management of schizophrenia, bipolar disorder, and other psychiatric conditions. However, AAPs are associated with metabolic syndromes (MetS) such as weight gain, dyslipidemia, type 2 diabetes (T2D), and hypertension, which can contribute to reduced life expectancy and poor medication adherence [7, 8].

Previous studies have highlighted a close relationship between the clinical efficacy of antipsychotic medications and their serum concentrations [9, 10]. Suboptimal serum levels may reduce clinical efficacy, while excessive concentrations increase the risk of adverse effects, such as extrapyramidal reactions [11]. Recent research has suggested that glucose and lipid metabolism disturbances play a critical role in the pathophysiology of SCH [12, 13]. Consequently, glucose and lipid metabolism alterations are considered important clinical efficacy markers. Lipid metabolism markers, including total cholesterol (TC), triglycerides (TG), high-density lipoprotein (HDL), and low-density lipoprotein (LDL), are key indicators of metabolic health, with abnormal levels indicating dyslipidemia [14].

Numerous studies have shown that amisulpride exerts a relatively milder effect on blood glucose and lipids than other antipsychotics, such as clozapine and risperidone [15, 16]. However, amisulpride plasma/serum levels exhibit significant inter-individual variability, with a significant proportion of patients having concentrations outside the recommended therapeutic range [17]. Therefore, monitoring the blood concentration of SCH patients is the basis of rational and efficient drug use. Despite extensive research abroad, the relationship between amisulpride serum concentrations, therapeutic efficacy, and side effects remains inconsistent across different studies, presenting a challenge for its clinical application in SCH treatment. This study aimed to monitor serum amisulpride concentrations in adult female SCH patients and analyze their influence on clinical efficacy, glycolipid metabolism, and side effects.

## Materials and Methods

### General Information

This retrospective study included female SCH patients admitted to the Third People’s Hospital of Yongkang City between January 2020 and January 2022. A total of 122 patients were treated with amisulpride monotherapy and were selected as the study cohort. Patients ranged in age from 23 to 56 years, with a mean age of 42.09 ± 8.46 years. The duration of SCH ranged from 1–10 years, with an average disease duration of 4.70 ± 1.66 years.

Inclusion criteria: (1) Female patients aged 18 years or older; (2) Diagnosis of schizophrenia based on the 3rd edition of the Chinese Criteria for Classification and Diagnosis of Mental Disorders [18], with primarily positive symptoms; (3) Disease duration of <10 years; (4) Informed consent from the guardian of the patient for study participation; (5) Ability to adhere to follow-up during the study period. Exclusion criteria: (1) Presence of serious organic or physical disease; (2) Known allergy to amisulpride or related compounds; (3) Medical conditions that could affect the efficacy of the drug; (4) History of alcohol dependence; (5) Previous treatment with two or more antipsychotic medications at the time of enrollment; (6) Significance self-harm violent tendencies; (7) Inability to comply with prescribed medication protocols.

This study was approved by the Medical Ethics Commission of the Third People’s Hospital of Yongkang (Ethics approval number: YKSY-2020-LC-12-08C1). Informed consent was obtained from all patients or their legal guardians, and the study was carried out in accordance with the Declaration of Helsinki.

### Methods

Patients discontinued original treatment regimens and initiated amisulpride therapy after a 1-week placebo washout period. The treatment protocol was as follows: oral amisulpride (H20113231, Qilu Pharmaceutical Co., Ltd., Jinan, China) was administered at an initial dose of 0.1 g/day, with increments of 0.1 g/day, gradually increasing to a total daily dose of 0.8–1.2 g/day over a 2-week period based on clinical response. Doses ≤0.4 g/day were administered in the evening, while doses >0.4 g/day were divided into morning and evening administrations. Additionally, low-dose benzodiazepines were prescribed to improve sleep quality, and anti-arrhythmic propranolol and extrapyramidal tablets were administered to manage extrapyramidal symptoms during the treatment period.

### Observation Indicators

(1) Blood concentration: Fasting venous blood samples were collected before treatment and at 1, 2, 4, and 8 weeks post-treatment, with measurements taken within 48 hours of collection. Serum amisulpride concentrations were analyzed using high-performance liquid chromatography (HPLC) with an Agilent 1100 HPLC system (Agilent Technologies, Waldbronn, Germany) equipped with a fluorescence detector and a high-speed centrifuge (75003530, Abbott Laboratories, Chicago, IL, USA). Chromatographic conditions included an XBridge®C18 (4.6 mm × 250 mm, 5 µm, Waters Technologies Inc., Dublin, Ireland) maintained at 35 °C, with sodium heptane sulfonate-acetic acid solution and ethanol as the mobile phase. Fluorescence detection was performed at a wavelength of 225 nm with a sample injection volume of 20 µL. Plasma was mixed with 20 µL of amisulpride stock solution and thoroughly mixed, followed by the addition of 100 µL ammonium acetate buffer (35 mL) and 3 mL of Job’s seed for extraction. After vortexing and centrifugation, the upper organic phase was separated, and ether extraction was repeated twice. The final supernatant was used for chromatographic analysis. Blank plasma (0.5 mL) spiked with amisulpride at 10, 400, and 1000 µg/mL concentration was used as the standard solution. The chromatographic integration was carried out using a dedicated software, yielding a standard curve equation of y = 0.1355x – 0.5038, R^2^ = 0.9999, demonstrating good linearity over the 51.55–2061.94 ng/mL range.

(2) Clinical efficacy: The Brief Psychiatric Rating Scale (BPRS) [19] was used to assess clinical symptoms at baseline and at 1, 2, 4 and 8 weeks post-treatment. The scale consists of 18 items assessing hostility, suspicion, anxiety, disorientation, and more, with each item scored from 1 to 7. Total scores were calculated by summing all items. Treatment efficacy was classified based on the percentage reduction in BPRS score from baseline: a reduction of ≥75% was considered a cure, 50%–74% a significant improvement, 25%–49% moderate improvement, and ≤25% as ineffective. The total efficiency rate was defined as the sum of the cure, significant improvement and moderate improvement rates.

(3) Blood glucose and lipid metabolism: Fasting venous blood samples were collected at baseline and at 1, 2, 4 and 8 weeks post-treatment to assess fasting blood glucose, total cholesterol (TC), triglycerides (TG), high-density lipoprotein (HDL), and low-density lipoprotein (LDL) levels. These parameters were measured using an automatic biochemical analyzer (BK-280, Shandong Broco Enterprise Co., Ltd., Jinan, China).

(4) Side effects: Adverse effects were assessed using the Treatment Emergent Symptom Scale (TESS) [20] to evaluate side effects such as hypotension, loss of appetite, nausea, and vomiting. Scores ranged from 1 to 4, with higher scores indicating more severe side effects.

All measurements and evaluations were performed by trained medical personnel.

### Statistical Methods

Data were analyzed using SPSS 21.0 (IBM, Armonk, NY, USA). The Kolmogorow-Smironov (K-S) test was used to analyze the normality of continuous variables, which are expressed as mean ± standard deviation ($\bar{x}$ ± s). One-way repeated measures analysis of variance (ANOVA) was used for comparisons across time points, with Greenhouse-Geisser correction applied if the assumption of the sphericity test was not satisfied. This was followed by Tukey’s post hoc test for multiple comparisons. The independent sample *t*-test was used for between-group comparisons. Categorical data are expressed as frequencies and percentages (n, %), and Pearson correlation analysis was used to assess relationships between variables. A *p*-value < 0.05 was considered statistically significant.

## Results

### Changes in Serum Amisulpride Concentration and BPRS Scores Before and After Treatment

The serum concentration of amisulpride increased in the 2nd, 4th, and 8th week of treatment compared to the first week (*p* < 0.05). Additionally, there was a significant reduction in BPRS scores at all time points post-treatment compared to those before treatment (*p* < 0.05), as shown in Table 1.

**Table 1.** **Changes in amisulpride concentration and BPRS scores before and after treatment ($\bar{x}$ ± s)**.

Time	Blood concentration (ng/mL)	BPRS score (points)
Pre-treatment	0	98.29 ± 4.61
End of 1st week after treatment	134.35 ± 30.35^a^	75.34 ± 4.63^a^
End of 2nd week after treatment	252.42 ± 38.10^a⁢b^	52.21 ± 3.67^a⁢b^
End of 4th week after treatment	354.99 ± 45.03^a⁢b⁢c^	41.12 ± 3.53^a⁢b⁢c^
End of 8th week after treatment	356.49 ± 47.49^a⁢b⁢c^	33.96 ± 2.92^a⁢b⁢c⁢d^
*F*-value	2072.173	5569.680
*p*-value	<0.001	<0.001

Note: BPRS, Brief Psychiatric Rating Scale; Compared with pre-treatment, ^a^*p* < 0.05; Compared with the end of 1st week after treatment, ^b^*p* < 0.05; Compared with the end of 2nd week after treatment, ^c^*p* < 0.05; Compared with the end of 4th week after treatment, ^d^*p* < 0.05.

### Correlation Between Serum Amisulpride Concentration and BPRS Scores

**Fig. 1.**
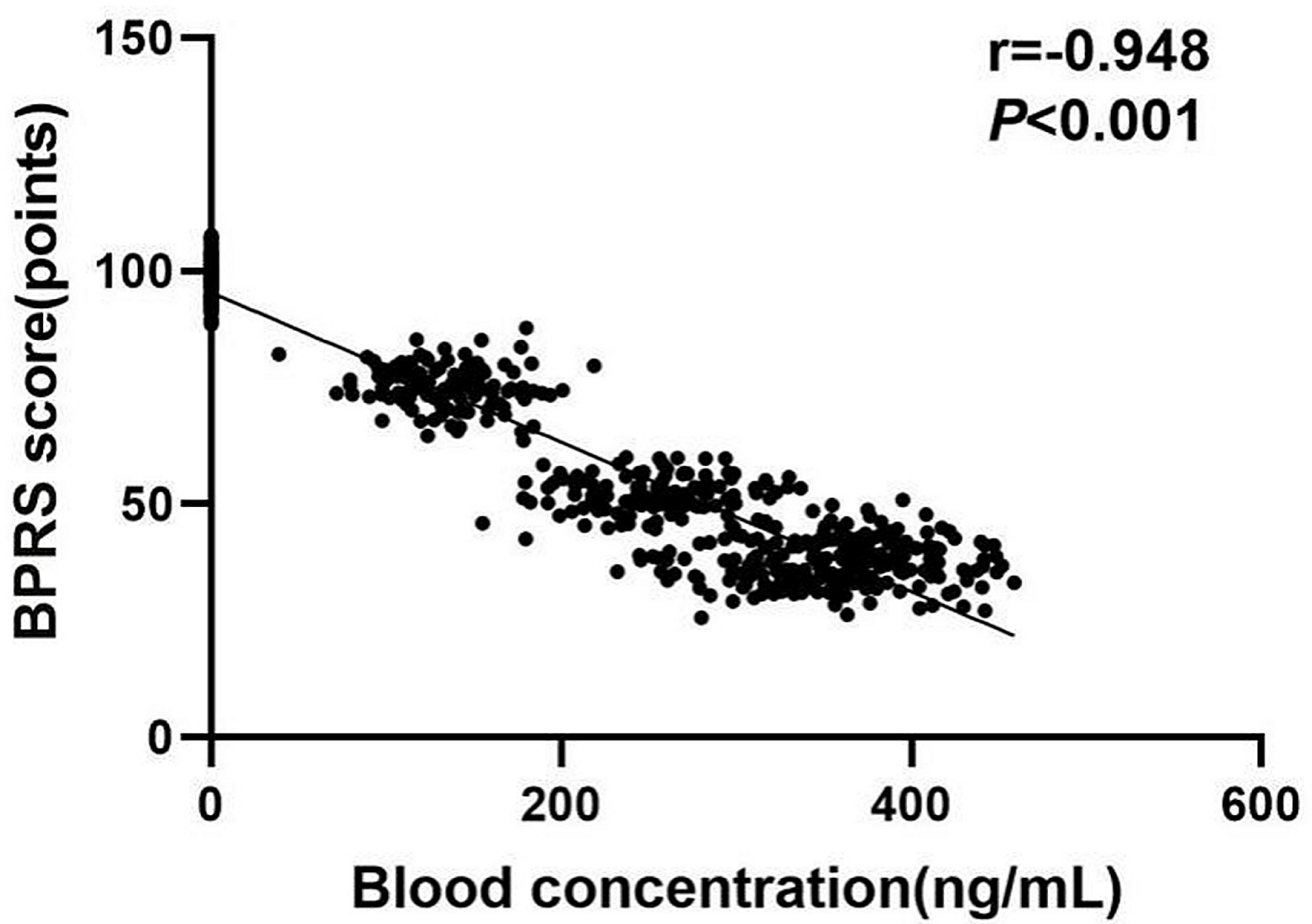
**A scatter plot showing the correlation between serum concentration of amisulpride and Brief Psychiatric Rating Scale (BPRS) score in schizophrenia (SCH) patients**.

A negative correlation was observed between amisulpride serum concentration and BPRS scores in SCH patients (r = –0.948, *p* < 0.001) (Fig. 1).

### Changes in Glycemic and Lipid Metabolism Indices Before and After Treatment

Significant changes in fasting blood glucose, TC, TG, HDL, and LDL levels were observed after treatment compared to before treatment (*p* < 0.05), as shown in Table 2.

**Table 2.** **Changes in glucose and lipid metabolism indices before and after treatment ($\bar{x}$ ± s, mmol/L)**.

Time	Fasting blood glucose	TC	TG	HDL	LDL
Pre-treatment	5.43 ± 0.75	4.51 ± 0.83	1.37 ± 0.11	1.41 ± 0.14	2.65 ± 0.20
End of 1st week after treatment	4.65 ± 0.42^a^	4.40 ± 0.50	1.44 ± 0.18^a^	1.55 ± 0.13^a^	2.43 ± 0.27^a^
End of 2nd week after treatment	4.61 ± 0.31^a^	4.20 ± 0.75^a⁢b^	1.49 ± 0.13^a⁢b^	1.42 ± 0.15^b^	2.13 ± 0.26^a⁢b^
End of 4th week after treatment	4.40 ± 0.27^a⁢b⁢c^	4.17 ± 0.57^a⁢b^	1.55 ± 0.16^a⁢b⁢c^	1.46 ± 0.17^a⁢b^	1.79 ± 0.14^a⁢b⁢c^
End of 8th week after treatment	4.21 ± 0.31^a⁢b⁢c⁢d^	4.31 ± 0.72^a⁢b^	1.60 ± 0.16^a⁢b⁢c⁢d^	1.44 ± 0.20^b^	1.87 ± 0.17^a⁢b⁢c⁢d^
*F*-value	130.876	5.422	44.119	15.824	352.330
*p*-value	<0.001	0.001	<0.001	<0.001	<0.001

Note: TC, total cholesterol; TG, triglycerides; HDL, high-density lipoprotein; LDL, low-density lipoprotein. Compared with pre-treatment, ^a^*p* < 0.05; Compared with the end of 1st week after treatment, ^b^*p* < 0.05; Compared with the end of 2nd week after treatment, ^c^*p* < 0.05; Compared with the end of 4th week after treatment, ^d^*p* < 0.05.

### Correlation Between Serum Amisulpride Concentration and Glycolipid Metabolism

**Fig. 2.**
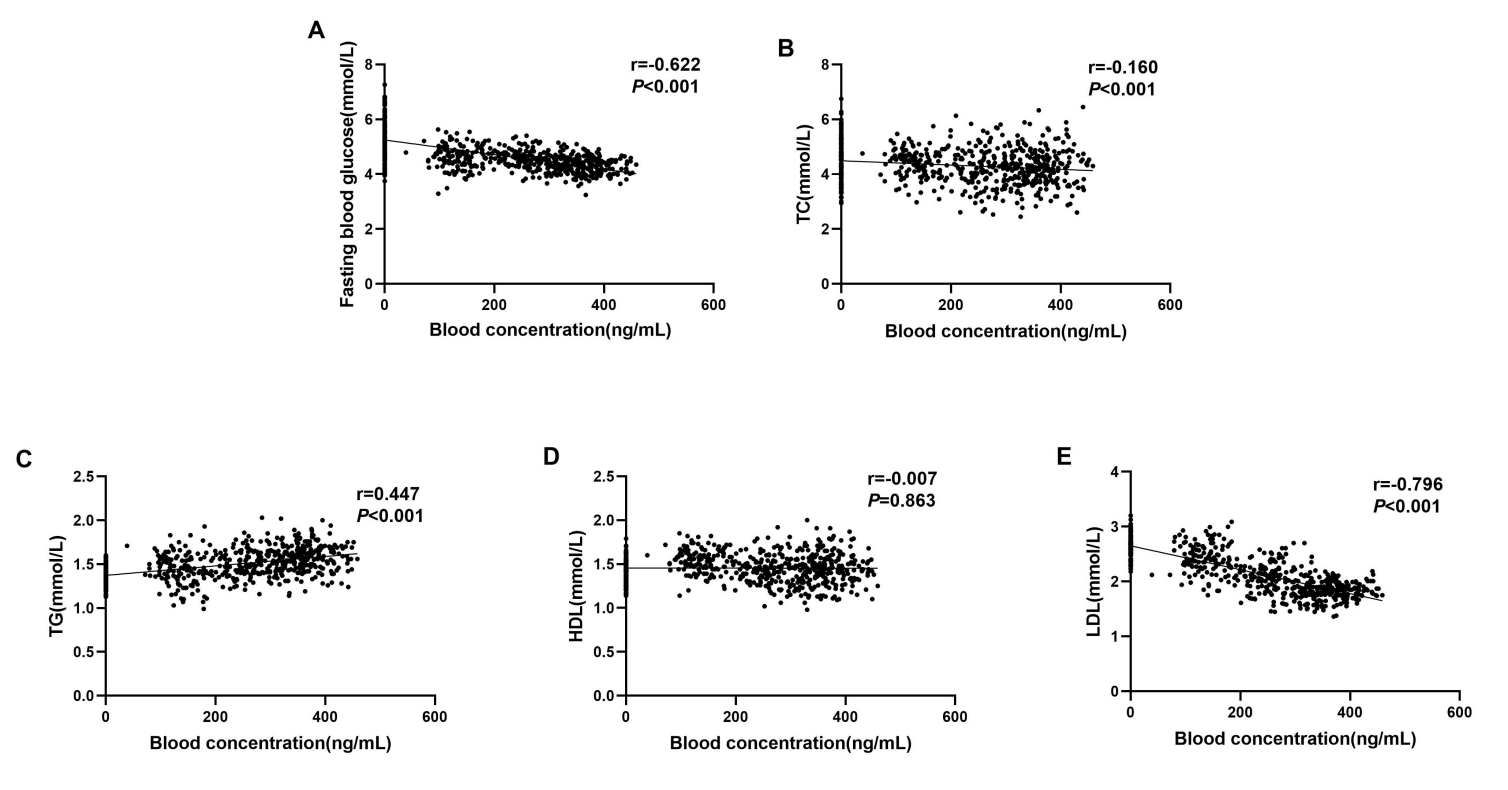
**Scatter plots (A–E) illustrating the correlations between amisulpride serum concentrations and metabolic markers in schizophrenia (SCH) patients**. (A) Fasting blood glucose, (B) total cholesterol (TC), (C) triglycerides (TG), (D) high-density lipoprotein (HDL), and (E) low-density lipoprotein (LDL).

Serum amisulpride concentration was negatively correlated with fasting blood glucose, TC and LDL levels (r = –0.622, –0.160, –0.796, respectively; *p* < 0.001) and positively correlated with TG levels (r = 0.447, *p* < 0.001). These correlations are detailed in Table 3 and illustrated in Fig. 2A–E.

**Table 3.** **Correlation between serum amisulpride concentration and glycolipid metabolism**.

Variable	r	*p*-value
Fasting blood glucose	–0.622	<0.001
TC	–0.160	<0.001
TG	0.447	<0.001
HDL	–0.007	0.863
LDL	–0.796	<0.001

Note: TC, total cholesterol; TG, triglycerides; HDL, high-density lipoprotein; LDL, low-density lipoprotein.

### Occurrence of Adverse Reactions

Adverse reaction scores increased significantly at the 2nd, 4th, and 8th weeks compared to the 1st week, with statistically significant differences in side effect scores at each time point (*p* < 0.05), as shown in Table 4.

**Table 4.** **Comparison of side effect scores after treatment ($\bar{x}$ ± s, points)**.

Time	Side effects score (points)
End of 1st week after treatment	1.57 ± 0.24
End of 2nd week after treatment	2.46 ± 0.39^a^
End of 4th week after treatment	2.75 ± 0.36^a⁢b^
End of 8th week after treatment	3.57 ± 0.51^a⁢b⁢c^
*F*-value	539.302
*p-*value	<0.001

Note: Compared with the end of 1st week after treatment, ^a^*p* < 0.05; Compared with the end of 2nd week after treatment, ^b^*p* < 0.05; Compared with the end of 4th week after treatment, ^c^*p* < 0.05.

### Correlation Between Serum Amisulpride Concentration and Side Effects Score

**Fig. 3.**
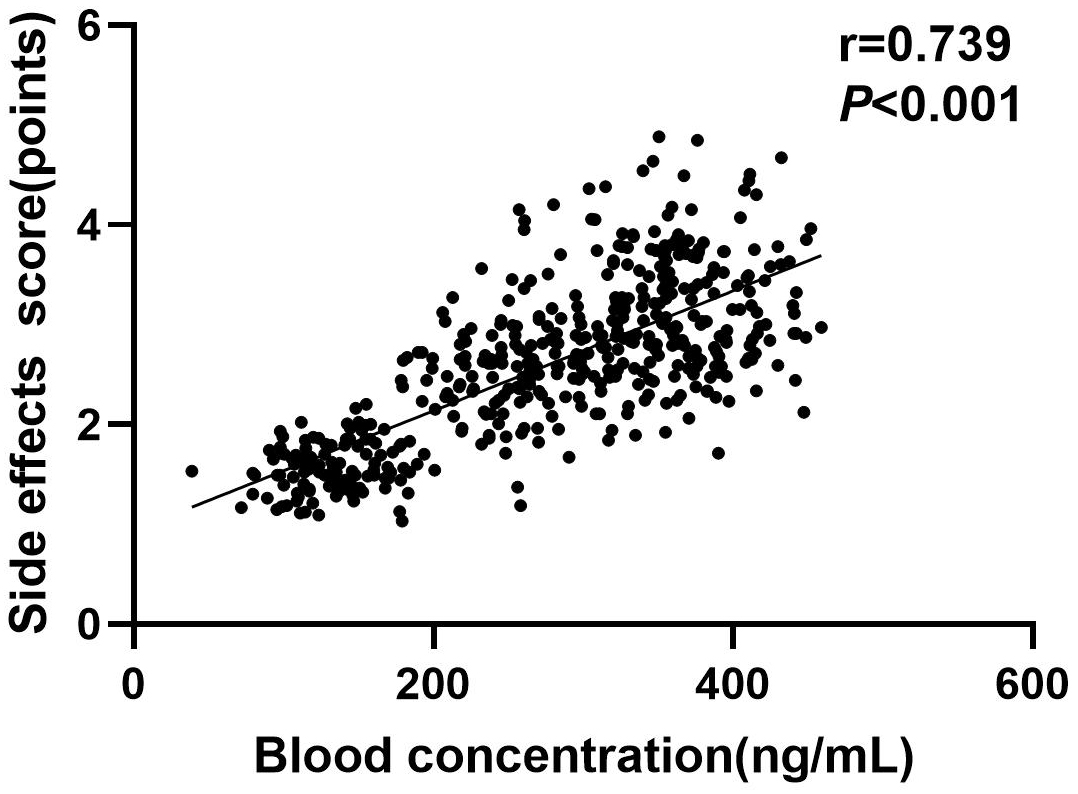
**Scatter plot demonstrating the correlation between serum concentration of amisulpride and side effects scores in schizophrenia (SCH) patients**.

A positive correlation was observed between serum amisulpride concentration and side effect scores in SCH patients (r = 0.739, *p* < 0.001) (Fig. 3).

## Discussion

Female schizophrenia (SCH) is prevalent in clinical practice, often necessitating antipsychotic treatment. Given the wide variety of available antipsychotics, it is necessary to select drugs with high efficacy and minimal side effects. Amisulpride, with its unique mechanism of action, has significantly improved positive, negative, and affective disorders in SCH patients. Due to its clinical benefits, amisulpride was classified as a Class I recommendation in the 2017 drug monitoring guidelines and is widely used in the treatment of SCH [21].

Currently, there is a growing focus on therapeutic drug monitoring, nationally and internationally. Studies have emphasized the significant relationship between drug dosage, blood drug concentration, and variable metabolic responses at the site of action [22, 23]. In this study, amisulpride concentrations were significantly higher at the 2nd, 4th, and 8th weeks of treatment compared to the 1st week, likely reflecting individual differences in drug metabolism. A steady-state concentration was observed by weeks 4 and 8. These findings are consistent with the results of Chang *et al*. [24], who also reported no significant difference in amisulpride blood concentration between weeks 4 and 8, suggesting that steady-state blood concentration was reached at the end of the fourth week of treatment. This stabilization occurs as the optimal therapeutic dose is achieved and the maintenance phase of treatment begins.

Our study also found a negative correlation between amisulpride serum concentration and BPRS score. In China, approximately 54.4% of patients have plasma levels higher than 320 ng/mL [25], highlighting the importance of monitoring blood concentration during amisulpride treatment to ensure therapeutic efficacy without risking adverse effects.

Previous research has demonstrated that abnormal glucose and lipid metabolism are closely related to SCH progression. Additionally, long-term antipsychotic treatment induces or exacerbates these metabolic abnormalities [26, 27]. Amisulpride is believed to have beneficial effects on glucose and lipid metabolism due to its potential to antagonize serotonin 2A receptors through the presynaptic D2 receptor and 5-hydroxytryptamine 1A (5-HT1A) receptors, thus helping to regulate metabolic balance [7]. In this study, fasting blood glucose, TC, TG, HDL, and LDL levels in SCH patients exhibited significant changes following amisulpride treatment compared with those before treatment. Correlation analysis revealed a significant relationship between fasting blood glucose, TC, TG and LDL levels and serum amisulpride concentration. These findings suggest that as serum amisulpride levels increase, its effect on regulating lipid metabolism in SCH patients also changes, highlighting the importance of maintaining serum concentrations within an appropriate range.

Further, our study revealed a significant correlation between amisulpride blood concentrations and therapeutic efficacy, consistent with findings by Sun *et al*. [28]. In therapeutic drug monitoring, it is necessary to assess the daily dosage and plasma amisulpride levels [25]. Currently, the Arbeitsgemeinschaft für Neuropsychopharmakologie und Pharmakopsychiatrie (AGNP) recommends monitoring amisulpride concentrations within a range of 100–320 ng/mL, with an alarm threshold of 640 ng/mL [29]. Despite adherence to dosage guidelines, clinical practice has shown that the blood drug levels of a patient may exceed the recommended range [3]. In treating positive schizophrenia symptoms, the starting dose typically ranges from 400–800 mg/day, while for predominantly negative symptoms, the starting dose is 50–300 mg/day. The stage of the mental disorder also determines the optimal drug concentration [30].

In this study, side effects scores increased at 1, 2, 4 and 8 weeks post-treatment, showing a positive correlation between serum amisulpride concentrations and the severity of side effects. These results suggest that as treatment duration extends, the risk of adverse reactions increases, in line with findings by Qu *et al*. [29]. Therefore, precise monitoring of amisulpride blood levels is essential for managing SCH treatment and mitigating side effects. In cases of significant side effects, combination therapy may be necessary to reduce amisulpride dosage while maintaining therapeutic efficacy. For SCH patients receiving amisulpride treatment, close monitoring of blood drug concentrations is essential to optimize dosing and guide individualized therapy. This study examined the relationship between amisulpride concentrations and therapeutic efficacy, glycolipid metabolism and adverse reactions in SCH patients. However, as a retrospective study, it is limited by its small sample size, single-center data, short observation period, and lack of consideration for the effect of enzyme metabolism. Future large-sample, multi-center clinical controlled studies are needed to account for individual differences due to amisulpride induction or inhibition and further investigate the mechanisms linking drug concentration, therapeutic effects, and lipid metabolism. Group clinical pharmacokinetics and pharmacodynamics studies are also warranted.

## Conclusion

In summary, the serum concentration of amisulpride in SCH patients is significantly correlated with therapeutic efficacy, lipid metabolism, and occurrence of side effects. Close monitoring of serum amisulpride levels is crucial in clinical practice to guide individualized treatment and optimize outcomes.

## Availability of Data and Materials

The data analyzed was available on the request for the corresponding author.
